# Effect of Probiotic Supplementation on Intestinal Permeability in Overweight and Obesity: A Systematic Review of Randomized Controlled Trials and Animal Studies

**DOI:** 10.1016/j.advnut.2023.100162

**Published:** 2023-12-08

**Authors:** Zachary DiMattia, Janhavi J Damani, Emily Van Syoc, Connie J Rogers

**Affiliations:** 1 Department of Nutritional Sciences, The Pennsylvania State University, University Park, PA, United States; 2 The Intercollege Graduate Degree Program in Integrative and Biomedical Physiology, Huck Institutes of Life Sciences, The Pennsylvania State University, University Park, PA, United States; 3 Integrative and Biomedical Physiology and Clinical and Translational Science, The Pennsylvania State University, University Park, PA, United States; 4 Department of Animal Science, The Pennsylvania State University, University Park, PA, United States; 5 The Microbiome Center, The Pennsylvania State University, University Park, PA, United States; 6 Department of Nutritional Sciences, College of Family and Consumer Sciences, University of Georgia, Athens, GA, United States

**Keywords:** obesity, probiotics, intestinal permeability, gut microbiome, inflammation, nutritional intervention, bifidobacterium spp, lactobacillus spp, akkermansia spp

## Abstract

Overweight and obesity are associated with increased intestinal permeability, characterized by loss of gut epithelial integrity, resulting in unregulated passage of lipopolysaccharide (LPS) and other inflammatory triggers into circulation, i.e., metabolic endotoxemia. In obesity, shifts in the gut microbiome negatively impact intestinal permeability. Probiotics are an intervention that can target the gut microbiome by introducing beneficial microbial species, potentially restoring gut barrier integrity. Currently, the role of probiotic supplementation in ameliorating obesity- and overweight-associated increases in gut permeability has not been reviewed. This systematic review aimed to summarize findings from both animal and clinical studies that evaluated the effect of probiotic supplementation on obesity-induced impairment in intestinal permeability (International Prospective Register of Systematic Reviews, CRD42022363538). A literature search was conducted using PubMed (Medline), Web of Science, and CAB Direct from origin until August 2023 using keywords of intestinal permeability, overweight or obesity, and probiotic supplementation. Of 920 records, 26 eligible records were included, comprising 12 animal and 14 clinical studies. Clinical trials ranged from 3 to 26 wk and were mostly parallel-arm (*n* = 13) or crossover (*n* = 1) design. In both animal and clinical studies, plasma/serum LPS was the most common measure of intestinal permeability. Eleven of 12 animal studies reported a positive effect of probiotic supplementation in reducing intestinal permeability. However, results from clinical trials were inconsistent, with half reporting reductions in serum LPS and half reporting no differences after probiotic supplementation. *Bifidobacterium*, *Lactobacillus*, and *Akkermansia* emerged as the most common genera in probiotic formulations among the animal and clinical studies that yielded positive results, suggesting that specific bacteria may be more effective at reducing intestinal permeability and improving gut barrier function. However, better standardization of strain use, dosage, duration, and the delivery matrix is needed to fully understand the probiotic impact on intestinal permeability in individuals with overweight and obesity.

**Statements of significance**

This systematic review summarizes the current state of knowledge on the efficacy of probiotic supplementation in reducing overweight- or obesity-associated increases in intestinal permeability. Probiotic genus, strain, dosage, length of supplementation, and delivery matrix were identified as important factors that may impact the ability of probiotics to reduce intestinal permeability in adults with overweight and obesity in both animal and clinical studies.

## Introduction

The prevalence of obesity in adults in the United States has risen from 30.5–42.4% over the past 18 y [1,2], despite many federal, state, and local initiatives to combat obesity [3,4]. This rising prevalence of obesity increases individual risk for other chronic diseases, as obesity and overweight are associated with multiple comorbidities, including type 2 diabetes, cardiovascular disease, and numerous types of cancer [[5], [6], [7]]. Adipose tissue plays a crucial role in energy homeostasis, wherein mobilization and storage of adipose triglycerides are under tight hormonal regulation [8,9]. Excessive macronutrient intake and metabolic dysfunction disrupt this energy homeostasis, promoting pathological expansion of visceral adipose tissue, including increased adipocyte hyperplasia and hypertrophy, and inducing hypoxia, necrosis, chemokine secretion, and uncontrolled fatty acid flux [[8], [9], [10], [11]]. This state leads to the production of monocyte chemoattractant protein-1, causing the infiltration of adipose tissue by circulating monocytes, which differentiate into macrophages that secrete various pro-inflammatory cytokines, including TNF-α, IL-1β, and IL-6, that drive chronic, low-grade systemic inflammation [10,[12], [13], [14]]. Obesity-associated chronic inflammation may have profound impacts on the gastrointestinal (GI) tract, including the maintenance of intestinal barrier integrity. In addition to elevated levels of pro-inflammatory markers, dyslipidemia, hyperglycemia, obese anthropometry, and the consumption of a Western-style diet also represent important risk factors for elevated intestinal permeability [[15], [16], [17]].

Intestinal permeability is regulated by tight junction proteins, such as occludin, tricellulin, claudins, junctional adhesion molecules, and Zonula occludens (ZO), which create paracellular spaces between enterocytes that selectively allow the flow of water and other small molecules into circulation [18,19]. However, under chronic disease conditions, these intercellular junction complexes can be disrupted, thus compromising intestinal barrier integrity and increasing the paracellular flux of larger molecules into circulation [[18], [19], [20]]. Increased intestinal permeability, also termed a “leaky gut,” results in the translocation of bacterial antigens, such as LPS, into the bloodstream, where circulating LPS binds LPS-binding protein (LBP) [21]. Circulating LPS from the LPS/LBP complex is recognized by a complex of Toll-like receptor 4 and myeloid differentiation factor 2 (MD-2) that are expressed on circulating immune cells [22]. Cluster of differentiation (CD) 14 facilitates the transfer of LPS to the Toll-like receptor 4/MD-2 complex on the immune cell surface, leading to downstream activation of Nuclear factor kappa-light-chain-enhancer of activated B cells (NF-κB)-mediated inflammatory cascades, thus contributing to chronic low-grade inflammation [19,22]. This condition of chronic but sub-clinical elevated plasma LPS is termed metabolic endotoxemia [23,24]. In mice, circulating LPS is a mediator of adipose tissue hypertrophy, inflammation, and metabolic disturbances [23,24]. Similar associations are observed in humans, where elevations in plasma LPS are associated with higher insulin insensitivity and inflammatory markers [19,25,26]. Increases in intestinal permeability and plasma LPS may contribute to the development of cardiovascular disease [27], metabolic dysfunction-associated fatty liver disease [28], and obesity [29].

Intestinal permeability is altered by intestinal dysbiosis, inflammatory cytokines, immune cell activation, and overall enterocyte health [17,18,[30], [31], [32], [33]]. The intestinal mucous layer is maintained by specialized epithelial cells known as goblet cells and serves a protective role against pathogens [31,34]. Pro-inflammatory cytokines, including interferon-γ and TNF-α, transiently increase intestinal permeability by inducing the rearrangement of tight junction proteins in the enterocyte membrane [18,35,36]. Zonulin is produced predominantly by epithelial cells in response to inflammatory signaling and is thought to act through protease-activated receptor 2 to phosphorylate ZO-1 in tight junctions and disrupt intercellular junction complexes [18,21,[35], [36], [37]]. Higher serum zonulin is associated with elevated waist circumference and increased risk of overweight and obesity, directly linking intestinal permeability to weight status [[38], [39], [40], [41]].

The gut microbiome also influences the relationship between obesity, inflammation, and intestinal permeability. The gut microbiome serves a critical role in fiber metabolism, anti-microbial protection, immune tolerance, and maintenance of the intestinal barrier [42]. Commensal bacteria produce short-chain fatty acids which provide energy for enterocytes and activate signaling pathways through G protein-coupled receptors, free fatty acid receptor 2, and free fatty acid receptor 3 in the intestines that stimulate 5-hydroxytryptamine, glucagon-like peptide-1 and peptide YY secretion [43,44]. In the GI tract, these hormones regulate colonic motility, inhibit gastric secretions, and reduce gut permeability [44,45]. Furthermore, the gut microbiome serves important metabolic roles that impact intestinal permeability, such as metabolizing ethanolamine, a molecule that reduces ZO-1 expression and disrupts tight junction complexes [46]. Gut microbial dysbiosis, a shift in intestinal bacterial composition that leads to a dysregulation of metabolic function for the host, can impact intestinal barrier integrity by altering these pathways [40,47]. Obesity is associated with decreased microbial diversity, altered pathways of bacterial energy metabolism, and a decrease in ethanolamine metabolism [46,48,49]. Probiotic supplementation may ameliorate obesity-induced gut microbial dysbiosis to restore gut homeostasis and improve intestinal barrier function [50,51].

Probiotics are microorganisms that, when administered in adequate amounts, confer a health benefit to the host [52]. The introduction of microbes through probiotics can contribute to transiently remodeling the gut microbiome to restore function in cases of dysbiosis [50,51]. Probiotic bacterial strains are capable of surviving transit through the upper GI tract and will colonize the gut during active supplementation, as quantified by the fecal presence of probiotic bacteria, but do not often persist long-term after cessation [53]. Major probiotic genera include *Bifidobacterium*, *Lactobacillus*, *Enterococcus*, *Streptococcus*, and *Saccharomyces* and are delivered in capsules, sachets, and food matrices [54]. Probiotic supplementation has been shown to ameliorate increased intestinal permeability in several animal models of GI disease [51]. Mechanistically, probiotics may promote tight junction protein production, increase mucus production, increase ethanolamine metabolism, and enhance enterocyte health through butyrate production [46,51]. The efficacy of probiotics to promote intestinal barrier integrity varies considerably by probiotic strains, delivery method, duration, population studied, and method of assessment [19,55].

Intestinal permeability in animal and clinical studies can be measured directly with functional tests that quantify the amount of a substrate that passes from the intestines into circulation via paracellular pathways or indirectly with biomarkers of intestinal barrier function or associated circulating inflammatory markers [19,25]. In functional tests, a nonabsorbable, nonmetabolizable tracer molecule is orally administered, and its concentrations are measured in blood or urine after a set time period. Commonly used tracers are sugars of varying molecular weights delivered as an oligosaccharide solution [25]. These sugars, including lactulose, mannitol, and sucralose, can be recovered to estimate site-specific intestinal permeability in the stomach, small intestine, and colon, depending on the size of the molecule and timing of collection [19,25]. Most commonly, the lactulose to mannitol ratio measured within the first 5 h after ingestion reflects small intestinal permeability, whereas sucralose and sucralose to mannitol ratio over the 5-to-24-h period after ingestion is an indicator of colonic permeability [56]. These tests, requiring only a urine sample, are used as a noninvasive assessment of intestinal permeability in clinical settings [25]. As alternatives to sugars, polyethylene glycols and fluorescein isothiocyanate (FITC)-dextran, a fluorescently labeled large sugar, are also used to assess intestinal permeability in rodent models functionally [25]. Healthy intestinal barriers are impermeable to these molecules, and their presence in blood and urine indicates increased barrier permeability [19].

Beyond functional tests, indirect indicators of intestinal permeability include biomarkers of bacterial translocation, inflammation, and/or enterocyte integrity [19]. Serum LPS and LBP are the most common clinical biomarkers of intestinal permeability with high specificity [19,25]. LPS is not found in large circulating quantities in healthy individuals, and leaky gut is the most common driver of non-sepsis increases in LPS [19,25]. Serum LPS and LBP are frequently measured using commercial ELISA kits or a Limulus Amebocyte Lysate assay [57]. Zonulin, measured in both the serum and stool [21], is another indirect marker of gut permeability as a potent endogenous modulator of enterocyte tight junctions [37]. Concentrations of serum anti-flagellin IgA and IgG can serve as emerging novel surrogate markers for gut permeability [58]. In animal models where tissue samples are readily available, intestinal tight junction protein concentrations or gene expression can also be measured directly [19,25,59,60]. Overall, various methods exist for assessing intestinal permeability both directly and indirectly, which necessitates thoughtful evaluation of these measurement methods when interpreting the therapeutic effects of probiotics on gut permeability [25].

Although probiotics are of clinical interest to target the obese gut microbiome for weight loss, limited attention has been given to intestinal permeability outcomes [19]. Current evidence indicates that the links between obesity, the intestinal microbiome, and intestinal permeability may profoundly influence metabolic health, which suggests that a leaky gut may be a therapeutic target [19,20]. Currently, the role of probiotic supplementation in ameliorating obesity- and overweight-associated increases in gut permeability has not been summarized. Thus, this review sought to summarize how probiotic dosage, genera, species, duration, delivery matrix, and measurement methods may impact obesity-related changes in intestinal permeability in animal and clinical studies.

## Methods

### Protocol registration and search strategy

This systematic review was conducted utilizing the PRISMA guidelines (Supplemental Table 1) [61] and was registered in PROSPERO (CRD42022363538) prior to article screening. A systematic literature search was conducted using PubMed (Medline), Web of Science, and CAB Direct from inception until August 2023 to identify studies that evaluate the effect of probiotic interventions on gut permeability outcomes in animal models of overweight or obesity and adults with overweight or obesity. A search strategy was developed in collaboration with a health science librarian at the Pennsylvania State University (CLW) and was comprised of 3 terms to represent: *1*) intestinal permeability, *2*) overweight or obesity, and *3*) probiotic supplementation. The full search terms used are found in the supplemental material (Supplemental Table 2).

### Inclusion and exclusion criteria

Only primary research articles written in the English language were included in this review. Inclusion criteria for both animal and clinical studies included subjects or animals with overweight or obesity, a probiotic intervention arm, and ≥1 measure of intestinal permeability. Animal studies investigating probiotics for the prevention of obesity in normal-weight animals were not included, as prevention was not the focus of this review. Studies that used additional interventions, such as prebiotics or prescribed medications, in conjunction with probiotics were included.

### Study selection, data extraction, and analysis

Complete searches of all 3 databases from origin through 08/31/2023 were conducted to obtain references for inclusion. A total of 920 references were exported, of which 375 duplicates were identified. After de-duplication, 545 references underwent a title and abstract screening independently by 2 investigators (ZD and JJD) to identify potentially eligible studies. The full texts of the identified studies were investigated independently with reference to the inclusion and exclusion criteria. The following information was extracted from each eligible study for analysis: bibliographic information (author and publication year), animal model or human subject characteristics, probiotic strains, probiotic dosage, length of intervention, colonization measurements, and intestinal barrier outcomes. Animal and clinical study results were summarized separately in tabular form based on intestinal permeability response to probiotic intervention.

### Quality assessment

For all animal interventions, quality was assessed using the Systematic Review Centre for Laboratory Animal Experimentation’s Risk of Bias (RoB) tool, which is based on the Cochrane RoB tool and designed for animal intervention studies [62]. Using this tool, quality is determined on 10 questions for the following 6 types of bias: selection bias, performance bias, detection bias, attrition bias, reporting bias, and other biases (Supplemental Table 3). In this table, a higher quantity of “yes” or “probably yes” answers are indicative of higher-quality studies. For all clinical interventions, quality was assessed using the second version of the Cochrane RoB2 tool with the crossover criteria where appropriate [63]. Quality was assessed based on entries for the following 5 domains of bias: randomization process, deviations from the intended interventions, missing outcome data, measurement of the outcome, and reported result (Supplemental Table 4). Crossover studies were assessed using the same domains and 1 additional domain on period and crossover (Supplemental Table 5). Using the flowchart guidance from Cochrane, each domain was assigned a RoB as “low,” “some concerns,” or “high,” and an overall risk was assigned based on those responses.

## Results

A total of 26 eligible studies, 12 animal and 14 clinical, were identified that quantified intestinal permeability outcomes with probiotic supplementation in animal or human populations with overweight or obesity. Results from each are summarized below.

### Findings from animal studies

Twelve studies investigated the effect of probiotic supplementation on gut permeability outcomes using animal models of obesity (Table 1) [[64], [65], [66], [67], [68], [69], [70], [71], [72], [73], [74], [75]]. Diet-induced obese (DIO) male C57BL/6J mice were used in 10 studies [[64], [65], [66], [67],[69], [70], [71], [72], [73], [74]], DIO male Kunming mice were used in 1 study [75], and DIO male Sprague Dawley rats were used in 1 study [68]. Obesity was induced in all animals using a high-fat diet prior to probiotic supplementation. Overall bias quality scores were assigned in Supplemental Table 3, with most studies scoring 7 out of 10 [[64], [65], [66], [67], [68],[70], [71], [72],^74^,^75^], and only 2 studies had scores lower than 6 [69,73].

**TABLE 1:** Findings from animal studies

Reference	Model	Probiotic supplementation	Dosage [delivery matrix]	Duration	Colonization	Intestinal permeability outcomes	
						Probiotic effect	Probiotic no effect
Bomhof et al., 2014 [68]	Obese male Sprague Dawley rats with DIO from HFD (*n* = 10/group)	*B. animalis* subsp. *lactis* BB-12	1 × 10^10^ CFU/d [unpurified diet]	8 wk	↑ fecal and cecal BB-12 (gene copies/ total DNA)	↑ *tjp1* gene expression (ileum)	*occludin* gene expression (ileum) FD-4 AUC Plasma LPS
Lim et al., 2017 [66]	Obese male C57BL/6 mice with DIO from HFD (*n* = 8/group)	*B. adolescentis* IM38	2 × 10^9^ CFU/d [gavage]	6 wk	Not measured	↓ plasma LPS ↑ ZO-1, occludin, and claudin-1 (colon)	None reported
Stenman et al., 2014 [73]	Male C57BL/6J mice with diabetes from HFD^1^ (increased fat mass) (*n* = 10/group)	*B. lactis* 420 (B420)	1 × 10^9^ CFU/d [gavage]	6 wk	Not measured	↓ plasma LPS ↓ mucosal adherence of gavaged *E. coli* (ileum and cecum)	Mucosal adherence of gavaged *E. coli* in duodenum and jejunum Translocation of *E. coli* to extra-intestinal tissues
In Kim et al., 2019 [72]	Obese male C57BL/6J mice with DIO from HFD (*n* = 10/group)	*L. plantarum* LC27 and *B. longum* LC67	1 × 10^9^ CFU/d [gavage]	4 wk	Not measured	↓ serum LPS ↑ occludin and claudin-1 (colon)	None reported
Heeney et al., 2019 [71]	Obese male C57BL/6J mice with DIO from HFD (*n* = 10/group)	*L. plantarum* WCFS1 (NCIMB8826-R)	5 × 10^8^ CFU/d [gavage]	9 wk	↑ fecal, cecal, and ileal BB-12 (gene copies as % of reads)	↑ ZO-1 (ileum)	*zo-1* gene expression (ileum)
Molina-Tijeras et al., 2021 [70]	Obese male C57BL/6J mice with DIO from HFD (*n* = 10/group)	*L. fermentum* CECT5716	5 × 10^8^ CFU/d [gavage]	11 wk	Not measured	↑ *muc-1, muc-2, muc-3, zo-1, occludin*, and *tff-3* gene expression (colon) ↑ occludin (colon) ↓ plasma LPS	None reported
Ashrafian et al., 2019 [64]	Obese male C57BL/6 mice with DIO from HFD (*n* = 5/group)	*A. muciniphila*	1 × 10^9^ CFU/d [gavage]	5 wk	Not measured	↑ mucosal thickness and crypt depth ↑ *zo-1, ocldn*, and *cldn-1* gene expression (colon) ↓ *cldn-2* gene expression (colon)	None reported
Everard et al., 2013 [69]	Obese male C57BL/6J mice with DIO from HFD (*n* = 6–10/group)	Live *A. muciniphila* OR pasteurized *A. muciniphila*	2 × 10^8^ bacterial cells/d [gavage]	4 wk	Not measured	↑ mucosal thickness - live *A. muciniphila* ↓ serum LPS - live *A. muciniphila*	Mucosal thickness - pasteurized *A. muciniphila* Serum LPS - pasteurized *A. muciniphila*
Cho et al., 2018 [65]	Obese male C57BL/6J mice with DIO from HFD (*n* = 10/group) (probiotic and synbiotic)	*L. mesenteroides* 4 (LCM4), and *L. kefiri* DH5 (DH5)	1 × 10^10^ CFU/d of *L. mesenteroides* 4, 1 × 10^9^ CFU/d of *L. kefiri* DH5 [gavage]	9 wk	Not measured	↓ plasma zonulin - synbiotic ↓ *hp* gene expression (adipocyte) - synbiotic ↓ LPS-binding protein (*lbp*) gene expression (adipocyte) - synbiotic	Plasma zonulin - probiotic Adipocyte *hp* gene expression - probiotic Adipocyte *lbp* gene expression - probiotic
Kwon et al., 2019 [67]	Obese male C57BL/6J mice with DIO from HFD (*n* = 8–10/group) (probiotic and synbiotic)	*L. mesenteroides* (DH 1604), and *L*. kefiri DH59	1 × 10^10^ CFU/d of *L. mesenteroides* 4, 1 × 10^9^ CFU/d of *L. kefiri* DH5 [gavage]	9 wk	Not measured	↓ plasma zonulin - synbiotic	Plasma zonulin - probiotic
de Moura e Dias et al., 2023 [74]	Obese male C57BL/6J mice with DIO from HFD (*n* = 7–8/group)	*L. gasseri* LG-G12 (LG-G12)	1 × 10^9^ CFU/d [gavage]	4 wk	Not measured	None reported	Urinary L/M ratio
Hu et al., 2023 [75]	Obese male Kunming mice with DIO from HFHSD (*n* = 8–10/group)	*L. plantarum* P101	2 × 10^8^ CFU/d [gavage]	2 wk	Not measured	*↑ cldn-1* gene expression (colon)	*zo-1* and *muc-2* gene expression (colon)

AUC, area under the curve; CFU, colony forming unit; DIO, diet-induced obesity; DNA, deoxyribonucleic acid; FD-4, fluorescein isothiocyanate-dextran 4,000 Da.; HFD, high-fat diet (60% kcal from fat); HFHSD, high-fat high sugar diet; *hp*, haptoglobin; L/M lactulose/mannitol; LPS, lipopolysaccharide; ZO, zonula occludens.

*A. muciniphila, Akkermansia muciniphila; B longum, Bifidobacterium longum; B. adolescentis*, *Bifidobacterium adolescentis; B. animalis, Bifidobacterium animalis; B. lactis, Bifidobacterium lactis; L. fermentum, Lactobacillus fermentum; L. gasseri, Lactobacillus gasseri; L. kefiri, Lactobacillus kefiri L. mesenteroides*, *Leuconostoc mesenteroides; L. plantarum*, *Lactobacillus plantarum; E. coli, Escherichia coli.*

1 Diabetic mouse model fed diet with 72% fat that experienced elevated fat mass without becoming fully obese.

The probiotics used in these 12 studies varied in strain, genus, dosage, and combination of strains. Individual strains and species are listed in Table 1 and were all from the genera *Akkermansia*, *Bifidobacterium*, *Lactobacillus*, and *Leuconostoc*. The dosage of probiotics varied by study, ranging from 1 x 10^8^ to 1 × 10^10^ colony forming unit (CFU)/animal/d, with a median dose of 1 × 10^9^ CFU/animal/d [interquartile range (IQR): 5 × 10^8^ – 4 × 10^9^]. Probiotic supplementation duration varied by study and ranged from 2 to 11 wk, with a median length of 6 wk (IQR: 4–9). Single-strain probiotic formulations were used in 9 studies [64,66,[68], [69], [70], [71],[73], [74], [75]], and multi-strain formulations were used in 3 [65,67,72]. Two studies used synbiotics, a combination of probiotics and prebiotics, in which catechin-rich wine grapeseed 4 was added as an adjuvant to probiotics [65,67]. Probiotics were delivered by oral gavage in 11 studies [[64], [65], [66], [67],[69], [70], [71], [72], [73]] and added to an unpurified diet in 1 [68]. Two studies measured colonization of probiotic species and reported an increase in gene copies of cecal, fecal, or ileal probiotic bacteria [68,71].

Gut permeability outcomes measured in the included studies were heterogeneous and included colonic tight junction gene expression (*n* = 4) [64,66,70,75]; colonic tight junction protein concentrations (*n* = 2) [70,72]; ileal tight junction gene expression (*n* = 2) [68,71]; ileal tight junction protein concentrations (*n* = 1) [71]; adipocyte gene expression (*n* = 1) [65]; plasma/serum LPS (*n* = 6) [66,[68], [69], [70],^72^,^73^]; fecal LPS (*n* = 1) [66]; plasma zonulin (*n* = 2) [65,67]; mucosal thickness (*n* = 2) [64,69]; mucosal adherence of Escherichia coli *(E. coli)* (*n* = 1) [73]; translocation of *E. coli* to extra-intestinal tissues (*n* = 1) [73]; intestinal permeability assessed by FITC dextran 4000 Da (*n* = 1) [73]; and intestinal permeability assessed by lactulose/mannitol ratio (*n* = 1) [74].

Eleven of the 12 animal studies reported a positive impact of probiotic supplementation on ≥1 measure of intestinal permeability. Serum/plasma LPS was reduced from baseline following probiotic supplementation with species of *Bifidobacterium*, *Lactobacillus,* or *Akkermansia* in 5 of 6 studies [66,69,70,72,73], with 1 study showing no effect of using *Bifidobacterium animalis* subspecies *lactis* BB-12 [68]. A single study reported a reduction in fecal LPS from baseline in mice supplemented with 2 × 10^9^ CFU/d of *Bifidobacterium adolescentis* IM38 for 6 wk compared to control [66]. Tight junction gene expression and protein concentrations measured in both the colon and ileum included *zo-1*, *ocln* (or *occludin*), *cldn-1*, *cldn-2*, *tjp1*, *muc-1*, *muc-2*, *muc-3*, *tff-3*, ZO-1, occludin, and claudin-1 [64,66,68,70,71,75]. Five studies assessed colonic gene expression of tight junction proteins, colonic tight junction protein concentrations, or both after probiotic supplementation with species of *Bifidobacterium*, *Lactobacillus,* or *Akkermansia* and reported both increases in the expression of ≥1 gene (*n* = 3) or protein concentrations (*n* = 3) [64,66,70,72,75]. Two studies assessed ileum gene expression of tight junction proteins, ileum tight junction protein concentrations, or both after probiotic supplementation with species of *Bifidobacterium* or *Lactobacillus* and reported mixed results of increases in *tjp1* gene expression (*n* = 1) [68] and ZO-1 protein concentrations (*n* = 1) [71] but no changes in *occludin* or *zo-1* gene expression (*n* = 2) [68,71]. Two studies reported a reduction in plasma zonulin from baseline after supplementation with *Lactobacillus kefiri* and *Leuconostoc mesenteroides* when a prebiotic was included with probiotic administration but not when probiotics were administered alone [65,67]. Two studies reported increases in mucosal thickness after supplementation of *Akkermansia muciniphila* for 4 to 5 wk in DIO mice compared to control (*n* = 2) [64,69]. One study examined adipocyte gene expression and found increases in the expression of zonulin (*hp*) and *lbp* when a prebiotic was included with supplementation of *Lactobacillus kefiri* and *Leuconostoc mesenteroides* for 9 wk but not when probiotics were administered alone [65]. A single study assessed the ability of gavaged *E. coli* to adhere to the mucosa in each intestinal region; following supplementation of 2 × 10^9^ CFU/d of *Bifidobacterium lactis* 420 for 6 wk, there was decreased *E. coli* adherence in the ileum and cecum, whereas the duodenum and jejunum were unaffected [73]. In this study, the translocation of *E. coli* to extra-intestinal tissues was unaffected by probiotic supplementation [73]. Two studies reported no effect on in vivo intestinal permeability as measured by the fluorescent-conjugated dye, FITC dextran 4000 Da [68] and the ratio of lactulose/mannitol [74] in DIO mice supplemented with 1 × 10^10^ CFU/d of *Bifidobacterium animalis* subspecies *lactis* BB-12 for 8 wk or 1 × 10^9^ CFU/d of *Lactobacillus gasseri* for 4 wk, respectively.

### Findings from clinical trials

Fourteen studies, 13 parallel-arm, and 1 crossover investigated the effect of probiotic supplementation on gut permeability outcomes in participants with overweight or obesity (Table 2) [[76], [77], [78], [79], [80], [81], [82], [83], [84], [85], [86], [87], [88], [89]]. Participants in most studies were 18–65 y old, with the exception of 3 studies that recruited older participants of 30+ [79], 45+ [76], and 50+ [83]. Two studies recruited exclusively female participants [76,87], whereas the rest recruited males and females randomly. Overweight or obese status was determined by BMI (in kg/m^2^) in all studies. A minimum BMI of 25 was used in 13 of 14 studies as the cutoff for overweight, except for a single study conducted in Thailand that used the Thai overweight threshold of BMI ≥20 [83]. Three studies recruited participants with a BMI specifically between 30–40 [76,78,82]. Inclusion and exclusion criteria regarding pharmaceutical usage varied; 3 studies excluded the use of any current prescriptions [77,80,84], and 7 studies excluded the use of specific drugs, including statins, antihypertensives, antidiabetics, anti-inflammatory drugs, and weight loss medications [76,79,81,85,86,88,89], and 4 studies did not specifically exclude pharmaceutical use [78,82,83,87]. Two studies investigated interactions between probiotics and anti-diabetic medications and recruited subsets of participants on metformin treatment or other antihyperglycemic agents [78,86]. Overall, RoB was assigned in Supplemental Tables 4 and 5. All studies had “low” RoB, except for 1 that was assigned to “some concerns” because of concerns with randomization and blinding [80].

**TABLE 2:** Findings from clinical studies

Reference	Study design	Subjects	Probiotic supplementation	Dosage [delivery matrix]	Duration	Colonization	Intestinal permeability outcomes	
							Probiotic effect	Probiotic no effect
Depommier et al., 2019 [84]	Parallel-arm RCT Control *n* = 11 Pasteurized *n* = 12 Live *n* = 9	Age 18–70, BMI >25kg/m^2^, (F = 17/M = 15)	Live *A. muciniphila* OR pasteurized *A. muciniphila*	1 × 10^10^ bacteria/d [PBS containing glycerol]	12 wk	↑ fecal *A. muciniphila* (CFU/g) - live > pasteurized	↓ serum LPS - pasteurized *A. muciniphila*	Serum LPS - live *A. muciniphila*
Szulińska et al., 2019 [76]	Parallel-arm RCT Control *n* = 27 Low dose *n* = 27 High dose *n* = 27	Age 45–70, ≥1 y after menopause, BMI 30–45kg/m^2^ (F = 81/M = 0)	Ecologic Barrier HD (*B. bifidum* W23, *B. lactis* W51, *B. lactis* W52, *L. acidophilus* W37, *L. brevis* W63*, L. casei* W56, *L. salivariu*s W24, *L. lactis* W19, and *L. lactis* W58)	1 × 10^10^ CFU/d (high dose), 2.5 × 10^9^ CFU/d (low dose) [sachet]	12 wk	Not measured	↓ serum LPS - high dose	Serum LPS - low dose
Liu et al., 2021 [81]	Parallel-arm trial 4 arms *n* = 20 to *n* = 23 (probiotic+low carb, low carb, AAD, low BMI)	Age 18–60, BMI ≥25kg/m^2^ (F = 45/M = 41)	*B. longum*, *L. acidophilus*, and *E. faecalis*	Combined 2.4 × 10^7^ CFU/d [capsule]	24 wk	Not measured	↓ serum LPS ↑ serum DAO ↓ serum D-lactic acid	None reported
Chaiyasut et al., 2022 [83]	Parallel-arm RCT Control *n* = 24 Intervention *n* = 24	Age ≥50, BMI ≥20kg/m^2^^1^ (F = 38/M = 10)	*L. paracasei* HII01, *B. breve*, and *B. longum*	Combined 5 × 10^10^ CFU/d [sachet]	12 wk	Not measured	↓ serum LPS ↓ urinary L/M ratio ↓ urinary lactulose % recovered	None reported
Chaiyasut et al., 2021 [85]	Parallel-arm RCT Control *n* = 36 Intervention *n* = 36	Age 18–65, BMI ≥25kg/m^2^^2^ (F = NR/M = NR)	*L. paracasei*, *B. longum*, and *B. breve*	Combined 5 × 10^10^ CFU/d [sachet]	12 wk	Not measured	↓ urinary lactulose % recovered ↓ urinary L/M ratio -suggested by Gaussian regression ↓ serum LPS ↓ serum ZO-1	Urinary L/M ratio
Krumbeck et al., 2018 [82]	Parallel-arm RCT 6 arms *n* = 17 to *n* = 20 (IVS-1, IVS-1 + GOS, BB-12, BB-12 + GOS, lactose, GOS)	Age 18–65, BMI 30.0–40.0 kg/m^2^ (F = 71/M = 23)	*B. adolescentis* IVS-1, OR *B. anima*lis ssp. *lactis* BB-12	1 × 10^9^ CFU/d [sachet]	3 wk	↑ RA fecal *B. adolescentis -* IVS-1, IVS-1 + GOS ↑ RA fecal *B. anima*lis - BB-12, BB-12 + GOS	↓ urinary S/L ratio - IVS-1, IVS-1 + GOS (w/aspirin) ↓urinary sucralose % recovered - BB-12 and GOS (w/aspirin)	Serum LPS Serum LBP Urinary S/L ratio (w/o aspirin) Urinary sucralose % recovered (w/o aspirin)
Horvath et al., 2020 [78]	Parallel-arm RCT Control *n* = 14 Intervention *n* = 12 (synbiotic)	Age ≥ 18, BMI 30–40 kg/m^2^ (F = 7/M = 19)	*B. bifidum* W23, *B. lactis* W51, *B. lactis* W52, *L. acidophilus* W37, *L. casei* W56, *L. brevis* W63, L. salivarius W24, *L. lactis* W58, and *L. lactis* W19	Combined 1.5 × 10^10^ CFU/d [sachet]	24 wk	*↑* fecal *L. brevis* in 8 of 12 subjects	↓ serum zonulin - 3 mo	Serum LPS - 3 or 6 mo Serum DAO Serum bacterial DNA serum LBP Serum sCD14
Pražnikar et al., 2020 [79]	Crossover RCT 2 period *n* = 28 (probiotic kefir, milk)	Age 30–60, BMI 25–29.9 kg/m^2^ (F = 15/M = 13)	*L. parakefiri*,*L. kefiri*,*L. kefiranofacie*nsssp. *Kefirgranum*, *R. kratochvilovae, K. marxianus*,and *K. exigua* delivered in kefir	300 mL kefir/d [food matrix]	3 wk	Not measured	↓ serum zonulin concentrations	Serum CRP Serum adiponectin
Palacios et al., 2021 [86]	Parallel-arm RCT Control *n* = 30 Intervention *n* = 30	Age ≥ 18, BMI >25kg/m^2^ (F = 32/M = 28)	*L. plantarum* Lp-115*, L. bulgaricus* Lb-64, *L. gasseri* Lg-36, *B. breve* Bb-03, *B. animalis* ssp. *lactis* Bi-07, *B. bifidum* Bb-06, *S. thermophilus* St-21, and *S. boulardii*	Combined 5 × 10^10^ CFU/d [capsule]	12 wk	*↑* RA fecal *B. breve, B. bifidum* No change in other probiotic bacteria	↓ plasma zonulin - subgroup taking metformin	Serum LPS Serum zonulin
Janczy et al., 2020 [80]	Parallel-arm RCT Control *n* = 20 Intervention *n* = 36 (synbiotic)	Age ≥ 18, BMI ≥25kg/m^2^ (F = 44/M = 12)	*B. lactis* W51, *B. lact*is W52, *L. acidophilus* W22, *L. paracasei* W20, *L. plantarum* W21, *L. salivarius* W24, and *L. lactis* W19	Combined 8 × 10^8^ CFU/d [capsule]	12 wk	*↑* fecal *Lactobacillus spp.* (CFU/g)	↓ fecal zonulin - synbiotic group	None reported
Leber et al., 2012 [88]	Parallel-arm RCT Control *n* = 15 Intervention *n* = 13	Age ≥ 18, BMI >25kg/m^2^, metabolic syndrome (F = 10/M = 18)	*L. casei* Shirota	1.95 × 10^10^ CFU/d [food matrix]	12 wk	Not measured	↑ serum LBP	Urinary saccharose % recovered Urinary L/M ratio Serum DAO Serum LPS
Lee et al., 2014 [87]	Parallel-arm RCT Control *n* = 25 Intervention *n* = 25	Age 19–65, BMI >25 kg/m^2^, WC >85 cm (F = 50/M = 0)	*S. thermophiles*, *L. plantarum*, *L. acidophilus*, *L. rhamnosus*, *B. lactis*, *B. longum,* and *B. breve*	Combined 1 × 10^11^ CFU/d [capsule]	8 wk	*↑* fecal *B. breve*, *B. lactis*, *L. rhamnosus*, *L. plantarum* No change in other probiotic bacteria	None reported	Serum LPS Urinary L/M ratio
Stevens et al., 2021 [77]	Parallel-arm RCT Control *n* = 34 Intervention *n* = 33	Age 18–70, BMI 25–35kg/m^2^ (F = 38/M = 29)	*B. indicus* P = D01	5 × 10^9^ CFU/d [sachet]	6 wk	*↑* fecal *B. indicus* (CFU/g)	None reported	Urinary 5 h L/R ratio 5–24 h S/E ratio 24 h S/E ratio
Kopp et al., 2023 [89]	Parallel-arm RCT Control *n* = 39 Intervention *n* = 37 (probiotic + omega-3s + vitamin D)	Age 25–65, BMI 28–40kg/m^2^ (F = 60/M = 16)	*L. paracasei (LCP-37), L. acidophilus (NCFM), B. lactis (Bi-07),* and *B. lactis (Bi-04)*	Combined 1 × 10^10^ CFU/d [capsule]	8 wk	Not measured	↓ Plasma I-FABP - females subgroup ↓ S/E ratio - females with obesity subgroup	5–19 h L/R ratio 5–24 h S/E ratio Urinary sucralose 0–24 h % recovered Plasma I-FABP Fecal zonulin

AAD, average American diet; BB-12, B. animalis ssp. lactis BB-12; BMI, body mass index; CFU, colony forming unit; DAO, diamine oxidase; DNA, deoxyribonucleic acid; F, female; GOS, galactooligosaccharide; I-FABP, intestinal-fatty acid-binding protein; IVS-1, B. adolescentis IVS-1; L/M lactulose/mannitol; L/R lactulose/rhamnose; LBP, lipopolysaccharide binding protein; LPS, lipopolysaccharide; M, male; NR, not reported; RA, relative abundance; RCT, randomized controlled trial; S/E, sucralose/erythritol; S/L, sucralose/lactulose; WC, waist circumference; ZO, zonula occludens; CRP, C-reactive protein ; PBS, Phosphate-buffered saline; sCD14.

*A. muciniphila, Akkermansia muciniphila; B. adolescentis*, *Bifidobacterium adolescentis*; *B. bifidum*, *Bifidobacterium bifidum; B. breve, Bifidobacterium breve; B. indicus, Bacillus indicus; B. lactis*, *Bifidobacterium lactis; B. longum, Bifidobacterium longum; E. faecalis, Enterococcus faecalis; K. exigua, Kazachstania exigua; K. marxianus, Kluyveromyces marxianus; L. acidophilus*, *Lactobacillus acidophilus; L. brevis, Lactobacillus brevis; L. bulgaricus, Lactobacillus bulgaricus*; *L. casei*, *Lactobacillus casei; L. gasseri, Lactobacillus gasseri*; *L. kefiranofaciens*, *Lactobacillus kefiranofaciens*; *L. kefiri, Lactobacillus kefiri; L. lactis*, *Lactococcus lactis; L. paracasei, Lactobacillus paracasei; L. parakefiri, Lactobacillus parakefiri*; *L. plantarum*, *Lactobacillus plantarum; L. rhamnosus, Lactobacillus rhamnosus; L. salivariu*s, *Lactobacillus salivarius; R. kratochvilovae, Rhodosporidium kratochvilovae; S. boulardii, Saccharomyces boulardii*; *S. thermophilus, Streptococcus thermophilus; S. thermophiles, Streptococcus thermophiles.*

1 Thai overweight adults.

2 Thai obese adults.

The probiotics used in these 14 studies varied in strain, genus, dosage, delivery matrix, and combination of strains. Individual strains and species are listed in Table 2 and were from the genera *Akkermansia*, *Bifidobacterium*, *Lactobacillus*, *Streptococcus*, *Lactococcus*, and *Bacillus*. The total dosage of probiotics varied by study, ranging from 2.4 × 10^7^ to 1 × 10^11^ CFU/person/d, with a median dose of 1 × 10^10^ CFU/person/d (IQR: 5 × 10^9^ – 5 × 10^10^). Probiotic supplementation duration varied by study and ranged from 3 to 24 wk with a median duration of 12 wk (IQR: 8–12). Single-strain probiotic formulations were used in 3 studies [77,84,88], and multi-strain formulations were used in 11 [76,[78], [79], [80], [81], [82], [83],[85], [86], [87],^89^]. Three studies used synbiotics and probiotics in combination with prebiotics, which primarily included galactooligosaccharides, fructooligosaccharides, and inulin [78,80,82]. Probiotics were delivered by sachet in 6 studies [[76], [77], [78],^82^,^83^,^85^], capsule in 5 studies [80,81,86,87,89], food matrix in 2 studies [79,88], and phosphate-buffered saline with glycerol in 1 study [84]. Seven studies measured probiotic colonization and viability and reported an increase in either relative abundance or gene copies of fecal probiotic bacteria during the trial supplementation period [77,78,80,82,84,86,87].

In clinical studies, there was variability in gut permeability outcomes measured, which included plasma/serum LPS (*n* = 10) [76,78,[81], [82], [83], [84], [85], [86], [87], [88]]; serum diamine oxidase (DAO) (*n* = 3) [78,81,88]; serum D-lactic acid (*n* = 1) [81]; serum LBP (*n* = 3) [78,82,88]; serum ZO-1 (*n* = 1) [85]; serum zonulin (*n* = 3) [78,79,86]; plasma intestinal-fatty acid-binding protein (*n* = 1) [89]; fecal zonulin (*n* = 2) [80,89]; and a mixed sugar solution consumption with urinary sugar analysis (*n* = 7) [77,82,83,85,[87], [88], [89]]. The types of sugars incorporated in mixed sugar solutions varied by study and included lactulose, mannitol, sucralose, saccharose, l-rhamnose, and erythritol. These studies calculated percent recovery and ratios of urinary lactulose, urinary lactulose/mannitol, urinary sucralose/lactulose, urinary sucralose, urinary saccharose, urinary lactulose/l-rhamnose, and urinary sucralose/erythritol to assess small and large intestinal permeability. Of the 7 studies that utilized mixed sugar solution urinalysis, the lactulose/mannitol ratio was the predominant choice (*n* = 4) [83,85,87,88].

Plasma/serum LPS was the most commonly measured biomarker in clinical studies (*n* = 10), among which 5 studies reported a reduction in LPS concentrations in adults after probiotic supplementation compared to placebo controls [76,81,[83], [84], [85]], whereas the other 5 studies reported no differences between interventions and controls [78,82,[86], [87], [88]]. Among these 10 studies, there was variability in bacterial genera, study duration, and probiotic dosage. A single study evaluated the dose-dependent effects of probiotic supplementation and reported that while the high-dose (1 × 10^10^ CFU/d) lowered serum LPS concentrations compared to the placebo, no differences were observed between the low-dose (2.5 × 10^9^ CFU/d) and placebo [76]. One study reported a reduction in serum DAO from baseline in adults overweight supplemented with 2.4 × 10^7^ CFU/d of *Bifidobacterium longum*, *Lactobacillus acidophilus*, and *Enterococcus* for 24 wk [81], whereas 2 other studies reported no differences between interventions and controls on serum DAO using 1.95 × 10^10^ CFU/d of *Lactobacillus casei* Shirota for 12 wk and a combined mix of 1.5 × 10^10^ CFU/d of *Bifidobacterium* and *Lactobacillus* for 24 wk [78,88]. A single study reported a reduction in serum D-lactic acid in adults with overweight supplemented with a combined 2.4 × 10^7^ CFU/d of *Bifidobacterium longum*, *Lactobacillus acidophilus*, and *Enterococcus faecalis* for 24 wk [81]. One study reported an increase in serum LBP from baseline in adults overweight supplemented with 2.4 × 10^7^ CFU/d of *Bifidobacterium longum*, *Lactobacillus acidophilus*, *Enterococcus faecalis* for 24 wk compared to control[88], whereas 2 other studies reported no differences between interventions and controls on serum LBP using 1.95 × 10^9^ CFU/d of *Bifidobacterium* for 3 wk and a combined 1.5 × 10^10^ CFU/d of *Bifidobacterium* and *Lactobacillus* for 24 wk [78,82].

A single study reported a decrease in serum ZO-1 from baseline in Thai adults with obesity supplemented with a combined 5 × 10^10^ CFU/d of *Lactobacillus paracasei*, *Bifidobacterium longum*, and *Bifidobacterium breve* for 12 wk compared to controls [85]. There was a reduction in serum zonulin from baseline in 2 studies on adults with overweight or obesity compared to controls [78,79], whereas another study reported no differences in serum zonulin between intervention and control except in a subgroup of patients taking metformin [86]. Fecal zonulin decreased from baseline in adults with overweight supplemented with a combination of *Bifidobacterium* and *Lactobacillus* at 8 × 10^8^ CFU/d for 12 wk when compared to controls [80], but another study with 1 × 10^10^ CFU/d of the same genera for 8 wk observed no differences between interventions and controls [89]. Plasma intestinal-fatty acid-binding protein was not impacted in the same study, except in females, which had significant reductions from baseline [89].

Only 1 study reported a decrease in urinary lactulose/mannitol ratio using Thai adults with overweight supplemented with a combined 5 × 10^10^ CFU/d of *Lactobacillus paracasei* HII01, *Bifidobacterium breve*, and *Bifidobacterium longum* for 12 wk [83] whereas 3 studies showed no differences in urinary lactulose/mannitol ratio between interventions and controls after probiotic supplementation [85,87,88]. One study reported no significant differences in lactulose/mannitol ratio between probiotic and placebo groups but noted that the magnitude of reduction was trending toward higher in the probiotic group (–0.11) than the placebo (–0.02) [85]. Two studies reported a reduction in the percent urinary lactulose recovered in Thai adults with overweight and obesity supplemented with 5 × 10^10^ of *Bifidobacterium* and *Lactobacillus* for 12 wk [83,85]. One study reported no differences between intervention and control on the ratio of sucralose/lactulose and the percent sucralose recovered in adults with obesity supplemented with either 1 x 10^9^ CFU/d *Bifidobacterium adolescentis* IVS-1 or 1 x 10^9^ CFU/d *Bifidobacterium anima*lis ssp. *lactis* BB-12 for 3 wk unless they received an aspirin challenge before the test [82]. However, 1 study demonstrated no change from baseline in urinary percent saccharose recovered in adults with overweight after 12-wk supplementation of 1.95 × 10^10^ CFU/d of *Lactobacillus casei* Shirota [88]. Furthermore, no differences between endpoints and baseline were reported in 2 studies on 5–19 h lactulose/l-rhamnose, 5–24 h sucralose/erythritol, 24 h sucralose/erythritol ratio, and sucralose percent recovered in adults with overweight and obesity after 6–8 wk of supplementation with 5 × 10^9^ CFU/d of *Bacillus indicus* or 1 × 10^10^ CFU/d of *Lactobacillus* and *Bifidobacterium* [77,89]. In a subgroup analysis, the sucralose/erythritol ratio of females with obesity was significantly decreased from baseline in comparison to placebo [89].

## Discussion

To date, no comprehensive review has summarized the effects of probiotic supplementation on intestinal permeability in adults with overweight and obesity. Given the relationship between increased intestinal permeability, metabolic risk markers, and visceral adiposity in humans [26,90,91], it is critical to evaluate how differences in probiotic species, formulations, dosages, duration, and assessment of intestinal permeability impact the efficacy of probiotics to improve gut barrier function in this population.

The majority of animal studies used serum/plasma LPS as an indicator of intestinal permeability [66,[68], [69], [70],^72^,^73^], of which 5 studies reported significant reductions in circulating LPS, regardless of differences in study design [66,69,70,72,73]. Urinary measurement of orally ingested tracer molecules is widely regarded as the standard in vivo measurement of intestinal barrier function in humans [92], and only 2 animal studies in the current review administered tracers [68,74]. In animal studies, the second most common method to assess gut barrier integrity was direct quantification of gene expression or protein concentrations of tight junction proteins. A previous review found that 15 of 17 animal studies on probiotics in mouse models of pathological intestinal conditions reported improvements in intestinal barrier functions as measured by upregulated tight junction (ZO-1, ZO-2, claudin-1, occludin, and junctional adhesion molecule-1) proteins, tight junction gene expression, and trans-epithelial electrical resistance [93]. These results align with the findings from the current review, where colonic tight junction protein concentrations and gene expression increased following probiotic supplementation in DIO mouse models [64,66,70,72,75]. However, we found heterogeneous results among the 2 studies that evaluated small intestinal tight junction proteins and gene expression. These data, specifically in the ileum, show that while there were increases in *tjp1* gene expression [68] and ZO-1 protein concentrations [71], there were no changes in *occludin* or *zo-1* gene expression [68,71]. This heterogeneity may be partially explained by the differential impact of probiotics on the gut microbiome in the small and large intestines, whereby the type and abundance of bacteria vary with changes in pH, digestive enzymes, and oxygen availability [94,95].

The majority of clinical studies used the assessment of serum/plasma LPS as a marker of intestinal permeability, whereas half of these studies reported a reduction in LPS following their highest probiotic consumption arm [76,81,[83], [84], [85]] and the other half reported no effect between the intervention and placebo arms [78,82,[86], [87], [88]]. A recent meta-analysis by Skonieczna-Zydecka et al. [96] evaluates 6 studies of healthy adults that supplemented probiotics and reports no impact on plasma LPS. However, healthy adults are less likely to have elevations in intestinal permeability and serum LPS prior to probiotic supplementation [88,97]; thus, these findings in a healthy population are not surprising. In contrast, 2 randomized controlled trials in unhealthy adults, 1 in participants with coronary artery disease and the second in patients undergoing gastric bypass surgery, demonstrate that supplementation for 12 wk with 1.6 × 10^9^ CFU/d of *Lactobacillus rhamnosus GG* or 16 wk with >10^10^ CFU/d of a multi-stain probiotic containing *Lactobacillus, Streptococcus*, and *Bifidobacterium* significantly decreased serum LPS and LBP compared to controls [98,99]. Although the majority of studies in the current review align with these results by showing reductions or no differences in serum LPS and LBP, there was a single randomized controlled trial [88] that reported significant increases in serum LBP after 12 wk of *Lactobacillus casei* Shirota supplementation in adults with metabolic syndrome. This unexpected finding may be because of the small sample size of this study (*n* = 28 total), but this probiotic strain should be investigated further in the context of increasing LPS translocation. Previous literature, in conjunction with our results, supports the hypothesis that probiotic supplementation of specific species may remediate increases in intestinal permeability and reduce LPS translocation in human subjects with inflammatory conditions [19,100].

Mixed sugar solution consumption, followed by urinary sugar analysis, was the second most common measure of clinical intestinal permeability in the current review [77,82,83,85,[87], [88], [89]]. A meta-analysis in patients with colorectal cancer by Liu et al. [101] evaluated the effect of probiotic supplementation in 4 studies on intestinal barrier integrity. In that population, probiotic supplementation for 6–17 d clinically decreased the lactulose/mannitol ratio, indicating a reduction in small intestinal permeability under inflammatory conditions [101]. The current review found 3 studies that report a reduction in the lactulose/mannitol ratio and lactulose percent recovered [82,83,85], and 4 studies showed no effect between the intervention and placebo arms [77,85,87,88]. This heterogeneity in outcomes is not unexpected since study designs differed in probiotic strain, dosage, and duration. The combination of strains from the genera *Bifidobacterium* and *Lactobacillus* [82,83,85] generally showed reductions in sugars recovered, whereas 2 studies using a single-strain *Bacillus indicus* P = D01 [77] and *Lactobacillus casei* Shirota [88] showed no effect. As with serum LPS and LBP, these findings indicate that specific probiotics may have a role in reducing intestinal permeability for populations that have prior elevations in intestinal permeability or intestinal damage.

In this review, the probiotic genera and strains varied considerably and were often in multi-strain formulations. Two previous reviews and a meta-analysis on the effects of probiotics on intestinal permeability in individuals with inflammatory responses such as colorectal cancer and cardiovascular disease show that the genera *Bifidobacterium* and *Lactobacillus* significantly decreased both serum zonulin and serum LPS [27,[100], [101], [102], [103]]. In assessing serum LPS alone, it should be considered that bacteria from the genera *Bifidobacterium* and *Lactobacillus* are gram-positive and could potentially alter intestinal LPS concentrations by replacing LPS-expressing gram-negative bacteria. The ability of these genera to improve the gut barrier is corroborated by functional measurements of intestinal permeability in these trials. Bacteria from these genera were utilized in 12 of the 14 clinical studies included in our review, with reductions in intestinal permeability mostly in multi-strain formulations [76,[78], [79], [80], [81], [82], [83],[85], [86], [87], [88]]. Our review supports the hypothesis that *Bifidobacterium* and *Lactobacillus* may significantly decrease intestinal permeability when used as a probiotic supplement in populations with inflammatory diseases. Beyond genera, the previous reviews suggest the bacterial strains *Lactobacillus plantarum*, *Lactobacillus acidophilus*-11, and *Bifidobacterium longum*-88 [[101], [102], [103]] induced reductions in serum zonulin and decrease in intestinal permeability. At least 1 of these species was utilized in 9 of the 14 clinical studies included in this review, largely in multi-strain formulations [76,78,80,81,83,[85], [86], [87],^89^]. The animal studies show similar patterns in genera, with *Bifidobacterium* and *Lactobacillus* being utilized in 10 of the 12 animal studies [[65], [66], [67], [68],[70], [71], [72], [73], [74], [75]] with mostly positive effects. Although *Bifidobacterium* and *Lactobacillus* are most widely used, the genus *Akkermansia* is being explored as another probiotic to reduce intestinal permeability. In the current review, findings from 2 animal studies and 1 randomized controlled trial suggest that *Akkermansia muciniphila* exerts positive effects on gut permeability by reducing serum LPS and increasing colonic tight junction gene expression [64,69,84]. Furthermore, *Akkermansia muciniphila* protects against increased LPS and restores gut barrier function in nonobese animal models [104,105]. Although the LPS-lowering effects reported with supplementation of Gram-positive probiotic species might be potentially confounded because of the lack of LPS expression by these strains, the reductions in serum LPS with supplementation of probiotics containing Gram-negative *Akkermansia muciniphilia* suggest that this species may reduce intestinal permeability by promoting enterocyte barrier function, thus reducing LPS translocation. Animal and human data together suggest that several species from the genus *Bifidobacterium*, *Lactobacillus*, and *Akkermansia* may be utilized to support intestinal barrier health in populations with impaired intestinal permeability.

Treatment duration and dosage are 2 other factors that impact the efficacy of probiotic supplementation. The minimum treatment length for the studies included was 2 and 3 wk for animal and clinical studies, respectively. The appropriate length for probiotic supplementation depends on the condition it is intended to treat, and current reports range from 1 to 5 d for acute diarrhea [106,107] and 2 mo to 2 y for dairy intolerance [108]. Although there are no studies that have evaluated the appropriate duration required for probiotic supplementation to impact intestinal permeability, previous meta-analyses report probiotic treatment durations from 6 d to 26 wk in trials where intestinal permeability is measured [100,101]. Probiotic dosage is another key factor that impacts probiotic efficacy. The minimum dosage used in the studies included were 1 x 10^8^ CFU/animal/d and 2.4 × 10^7^ CFU/person/d for animal and clinical studies, respectively. There is no minimum number of viable probiotic cells required to improve health outcomes, but doses between 10^8^ and 10^10^ CFU/d are reported to elicit beneficial effects for many probiotic interventions [109,110]. Regardless, 1 study in this review administered probiotics below 10^8^ CFU/d and still demonstrated reductions in serum LPS, which may be because of the longer intervention length of 24 wk [81]. Although some commercial probiotics contain 10^8^ – 10^10^ CFU/d, these products are intended to be consumed for only several weeks and may be inadequate to address increased intestinal permeability, depending on which strains are used [111]. One commercially available product with clinical usage, VSL#3, is dosed at 1 × 10^11^ – 9 × 10^11^ CFU/person/d [112], which is significantly higher than most commercial probiotics but has been shown to reduce intestinal permeability in both mouse models of colitis and adults with irritable bowel syndrome [[112], [113], [114], [115]]. Subsequently, not all treatment durations and dosages seen in this review may have been sufficient to impact intestinal permeability.

The current review is limited by heterogeneity in probiotic strains, duration, and dosage. It is difficult to assess if studies with no differences in measures of intestinal permeability can be attributed to a true lack of probiotic efficacy, short treatment durations, low dosages, or a combination of these factors. Another limitation is the heterogeneity in functional tests of intestinal permeability between animal and clinical studies. The variations in these testing methods complicate the translation of findings from animal research to clinical outcomes. Additionally, the included animal studies only used male animals, thus providing no insight into the impact of probiotic supplementation in female animals and potential sex differences. Two clinical studies that exclusively enrolled female participants [76,87] reported mixed results on reductions in serum LPS. These findings highlight the insufficiency of evidence to ascertain whether sex impacts how probiotic supplementation alters gut permeability. Thus, additional studies are needed to address this question. Furthermore, most clinical studies included in the review evaluated probiotic supplementation for <6 mo, thus making it difficult to draw any conclusions on the long-term effects of these interventions. Lastly, the use of multi-strain probiotic formulations limits our understanding of individual strain-specific effects of probiotics on intestinal barrier function in the context of overweight and obesity [116]. Future directions for this research should include comparisons of specific probiotic species on intestinal permeability in inflammatory diseases and additional studies to determine appropriate doses and treatment durations to reduce intestinal permeability in both sexes.

In conclusion, findings from animal studies suggest that individual administration of specific single-strain-species of *Bifidobacterium*, *Lactobacillus*, and *Akkermansia* for >2 wk may reduce intestinal permeability, as measured by plasma LPS and colonic tight junction gene expression, in animal models of obesity. Findings from clinical studies suggest that supplementation of probiotic formulations containing multi-strain bacteria from the genera *Bifidobacterium*, *Lactobacillus*, and *Akkermansia* for >3 wk may reduce intestinal permeability, as measured by plasma LPS or urinary sugar excretion, thereby restoring intestinal barrier function in adults with overweight or obesity. However, future studies with better standardization of strain use, dosage, duration, and delivery matrix are warranted to gain a comprehensive understanding of the strain-specific effects of probiotics on intestinal permeability in this population. Together, animal and clinical studies support the notion that probiotic supplementation can have a beneficial impact on intestinal permeability in an overweight or obese population.

## Author contributions

The authors’ responsibilities were as follows– CJR and ZD: designed the research; ZD and JJD: conducted the research and collected data; ZD: participated in data analysis and interpretation; ZD and CJR: wrote the manuscript; EVS: edited the manuscript; CJR: had primary responsibility for the final content, and all authors: read and approved the final manuscript.

## Conflict of interest

The authors report no conflicts of interest.

## Funding

ZD and JJD were funded by the National Center for Advancing Translational Sciences of the NIH under award number TL1TR002016. The content is solely the responsibility of the authors and does not necessarily represent the official views of the NIH. EVS was supported by AFRI Predoctoral Fellowship (grant no. 2022-67011-36461) and CJR was supported by grant no. 2017-67017-26780 from the USDA National Institute of Food and Agriculture. The funding agencies had no role in the study conceptualization, design, data collection, analysis, writing, decision to publish, or preparation and submission of the manuscript.
