# Supplementary material for: Effect of Probiotic Supplementation on Intestinal Permeability in Overweight and Obesity: A Systematic Review of Randomized Controlled Trials and Animal Studies
Source: Adv Nutr. 2023 Dec 8;15(1):100162. doi: 10.1016/j.advnut.2023.100162 (PMC10771892; doi:10.1016/j.advnut.2023.100162)
Supplement: Multimedia component 1 [file mmc1.docx]

**Supplemental Table 1.** Preferred Reporting Items for Systematic Reviews and Meta-Analyses (PRISMA) Checklist

| **Section/topic** | **#** | **Checklist item** | **Reported on page #** |
| --- | --- | --- | --- |
| **TITLE** | | |  |
| Title | 1 | Identify the report as a systematic review, meta-analysis, or both. | 1 |
| **ABSTRACT** | | |  |
| Structured summary | 2 | Provide a structured summary including, as applicable: background; objectives; data sources; study eligibility criteria, participants, and interventions; study appraisal and synthesis methods; results; limitations; conclusions and implications of key findings; systematic review registration number. | 2-3 |
| **INTRODUCTION** | | |  |
| Rationale | 3 | Describe the rationale for the review in the context of what is already known. | 4-8 |
| Objectives | 4 | Provide an explicit statement of questions being addressed with reference to participants, interventions, comparisons, outcomes, and study design (PICOS). | 8-9 |
| **METHODS** | | |  |
| Protocol and registration | 5 | Indicate if a review protocol exists, if and where it can be accessed (e.g., Web address), and, if available, provide registration information including registration number. | 9 |
| Eligibility criteria | 6 | Specify study characteristics (e.g., PICOS, length of follow-up) and report characteristics (e.g., years considered, language, publication status) used as criteria for eligibility, giving rationale. | 9-10 |
| Information sources | 7 | Describe all information sources (e.g., databases with dates of coverage, contact with study authors to identify additional studies) in the search and date last searched. | 9-10 |
| Search | 8 | Present full electronic search strategy for at least one database, including any limits used, such that it could be repeated. | Supplemental 3 |
| Study selection | 9 | State the process for selecting studies (i.e., screening, eligibility, included in systematic review, and, if applicable, included in the meta-analysis). | 9-10 |
| Data collection process | 10 | Describe method of data extraction from reports (e.g., piloted forms, independently, in duplicate) and any processes for obtaining and confirming data from investigators. | 10 |
| Data items | 11 | List and define all variables for which data were sought (e.g., PICOS, funding sources) and any assumptions and simplifications made. | 9 |
| Risk of bias in individual studies | 12 | Describe methods used for assessing risk of bias of individual studies (including specification of whether this was done at the study or outcome level), and how this information is to be used in any data synthesis. | 11 |
| Summary measures | 13 | State the principal summary measures (e.g., risk ratio, difference in means). | N/A |
| Synthesis of results | 14 | Describe the methods of handling data and combining results of studies, if done, including measures of consistency (e.g., I^2^) for each meta-analysis. | N/A |
| Risk of bias across studies | 15 | Specify any assessment of risk of bias that may affect the cumulative evidence (e.g., publication bias, selective reporting within studies). | N/A |
| Additional analyses | 16 | Describe methods of additional analyses (e.g., sensitivity or subgroup analyses, meta-regression), if done, indicating which were pre-specified. | N/A |
| **RESULTS** | | | |
| Study selection | 17 | Give numbers of studies screened, assessed for eligibility, and included in the review, with reasons for exclusions at each stage, ideally with a flow diagram. | 10 |
| Study characteristics | 18 | For each study, present characteristics for which data were extracted (e.g., study size, PICOS, follow-up period) and provide the citations. | 36-40 |
| Risk of bias within studies | 19 | Present data on risk of bias of each study and, if available, any outcome level assessment (see item 12). | 11-12,15 |
| Results of individual studies | 20 | For all outcomes considered (benefits or harms), present, for each study: (a) simple summary data for each intervention group (b) effect estimates and confidence intervals, ideally with a forest plot. | 12-19 |
| Synthesis of results | 21 | Present results of each meta-analysis done, including confidence intervals and measures of consistency. | N/A |
| Risk of bias across studies | 22 | Present results of any assessment of risk of bias across studies (see Item 15). | N/A |
| Additional analysis | 23 | Give results of additional analyses, if done (e.g., sensitivity or subgroup analyses, meta-regression [see Item 16]). | N/A |
| **DISCUSSION** | | | |
| Summary of evidence | 24 | Summarize the main findings including the strength of evidence for each main outcome; consider their relevance to key groups (e.g., healthcare providers, users, and policy makers). | 19-23 |
| Limitations | 25 | Discuss limitations at study and outcome level (e.g., risk of bias), and at review-level (e.g., incomplete retrieval of identified research, reporting bias). | 24 |
| Conclusions | 26 | Provide a general interpretation of the results in the context of other evidence, and implications for future research. | 24-25 |
| **FUNDING** | | | |
| Funding | 27 | Describe sources of funding for the systematic review and other support (e.g., supply of data); role of funders for the systematic review. | 1 |

**Supplemental Table 2.** Database, Search Terms, Filters, and Number of Results

| **Database**  **(Search Date)** | **Search Terms** | **Filters** | **Number of results** |
| --- | --- | --- | --- |
| PubMed  (08/31/2023) | (("Permeability"[MeSH Terms] OR "Permeabilities"[All Fields] OR "Permeability"[All Fields] OR "Leaky Gut"[All Fields] OR "Barrier"[All Fields]) AND ("Obesity"[MeSH Terms] OR "Obesity"[All Fields] OR "obese"[All Fields] OR "overweight"[MeSH Terms] OR "overweight"[All Fields] OR "over-weight"[All Fields] OR "over-weight"[All Fields]) AND ("probiotics"[MeSH Terms] OR "probiotics"[All Fields] OR "probiotic"[All Fields])) AND ((english[Filter])) | - | 286 |
| CAB Direct (8/31/2023) | (("obesity" OR "overweight" OR "obese" OR "over-weight" OR "over weight") AND ("probiotic" OR "probiotics") AND ("permeability" OR "permeabilities" OR "leaky gut" OR "barrier") | Language: English | 165 |
| Web of Science (WOS)  (8/31/2023) | ("obesity" OR "overweight" OR "obese" OR "over-weight" OR "over weight") AND ("probiotic" OR "probiotics") AND ("permeability" OR "permeabilities" OR "leaky gut" OR "barrier") | Language: English | 469 |

**Supplemental Table 3.** Assessment of Study Quality and Risk of Bias of Animal Interventions According to SYRCLE’s RoB Tool

| **Reference** | **Q1** | **Q2** | **Q3** | **Q4** | **Q5** | **Q6** | **Q7** | **Q8** | **Q9** | **Q10** | **Quality Score** |
| --- | --- | --- | --- | --- | --- | --- | --- | --- | --- | --- | --- |
| Bomhof *et al*., 2014 | PY | Y | PY | Y | NI | NI | NI | Y | Y | Y | 7 |
| Lim *et al*., 2017 | PY | Y | PY | PY | NI | NI | NI | Y | Y | Y | 7 |
| Stenman *et al*., 2014 | NI | PY | NI | NI | NI | NI | NI | Y | Y | Y | 4 |
| In Kim *et al*., 2019 | PY | Y | PY | PY | NI | NI | NI | Y | Y | Y | 7 |
| Heeney *et al*., 2019 | PY | Y | PY | PY | NI | NI | NI | Y | Y | Y | 7 |
| Molina-Tijeras *et al*., 2021 | PY | PY | PY | Y | NI | NI | NI | Y | Y | Y | 7 |
| Ashrafian *et al*., 2019 | PY | PY | PY | PY | NI | NI | NI | Y | Y | Y | 7 |
| Everard *et al*., 2013 | NI | PY | NI | NI | NI | NI | NI | Y | Y | Y | 4 |
| Cho *et al*., 2018 | PY | PY | PY | PY | NI | NI | NI | Y | Y | Y | 7 |
| Kwon *et al*., 2019 | PY | PY | PY | PN | NI | NI | NI | Y | Y | Y | 6 |
| de Moura e Dias *et al*., 2023 | PY | PY | PY | PY | NI | NI | NI | Y | Y | Y | 7 |
| Hu *et al*., 2023 | PY | PY | PY | PY | NI | NI | NI | Y | PY | Y | 7 |

Abbreviations: Y, yes; PY, probably yes; N, no; PN, probably no; NI, no information; NA, not applicable

Quality of included animal studies was assessed using the SYstematic Review Centre for Laboratory animal Experimentation (SYRCLE) RoB tool for animal intervention studies. Q1. Was the allocation sequence adequately generated and applied? Q2. Were the groups similar at baseline or were they adjusted for confounders in the analysis? Q3. Was the allocation adequately concealed? Q4. Were the animals randomly housed during the experiment? Q5. Were the caregivers and/or investigators blinded from knowledge of which intervention each animal received during the experiment? Q6. Were animals selected at random for outcome assessment? Q7. Was the outcome assessor blinded? Q8. Were incomplete outcome data adequately addressed? Q9. Are reports of the study free of selective outcome reporting? Q10. Was the study apparently free of other problems that could result in high risk of bias?

**Supplemental Table 4.** Assessment of Study Quality and Risk of Bias of Human Parallel Arm Interventions According to Cochrane RoB2 Tool

| **Reference** | | Szulińska *et al*., 2019 | Stevens *et al*., 2021 | Horvath *et al.*, 2020 | Janczy *et al.,* 2020 | Liu *et al*., 2021 | Krumbeck *et al*., 2018 |
| --- | --- | --- | --- | --- | --- | --- | --- |
| Randomization | Sequence generation random | Y | Y | Y | PY | Y | Y |
|  | Sequence concealment | Y | Y | PY | NI | Y | Y |
|  | Baseline Differences | N | N | N | PN | N | N |
|  | Risk of Bias | LOW | LOW | LOW | SOME CONCERNS | LOW | LOW |
| Deviations | Participant Aware of Assignment | N | N | N | PY | N | N |
|  | Researcher Aware of Assignment | N | N | Y | NI | N | N |
|  | Any Deviations | NA | NA | N | NI | NA | NA |
|  | Deviations Impact | NA | NA | NA | NA | NA | NA |
|  | Deviations Balanced | NA | NA | NA | NA | NA | NA |
|  | Risk of Bias | LOW | LOW | LOW | LOW | SOME CONCERNS | LOW |
|  | Appropriate analysis | N | N | NI | Y | N | NI |
|  | Appropriate Analysis Impact | PN | PN | PN | PN | NA | PN |
|  | Risk of Bias | SOME CONCERNS | SOME CONCERNS | SOME CONCERNS | SOME CONCERNS | SOME CONCERNS | SOME CONCERNS |
| Missing Outcomes | Data Availability All Participants | PN | PN | N | Y | N | PY |
|  | Non-bias by missing data | PY | PY | PY | NA | NI | NA |
|  | Could Missingness Depend on Value | NA | NA | NA | NA | PN | NA |
|  | Likely Missingness Depend | NA | NA | NA | NA | NA | NA |
|  | Risk of Bias | LOW | LOW | LOW | LOW | LOW | LOW |
| Measurement | Method Inappropriate | N | N | N | N | N | N |
|  | Method Differ Between Groups | N | N | N | N | N | N |
|  | Assessors Awareness | PN | NI | NI | NI | NI | N |
|  | Could Awareness Influence | NA | PN | PN | PN | PN | NA |
|  | Likely Awareness Influence | NA | NA | NA | NA | NA | NA |
|  | Risk of Bias | LOW | LOW | LOW | LOW | LOW | LOW |
| Reporting Bias | Analyzed with Pre-specified Plan | PY | Y | PY | PY | Y | Y |
|  | Selected with Multiple Outcome | PN | N | PN | PN | NI | PN |
|  | Selected with Multiple Analyses | PN | N | PN | PN | NI | PN |
|  | Risk of Bias | LOW | LOW | LOW | LOW | SOME CONCERNS | LOW |
| Overall Risk | Risk of Bias Judgement | LOW | LOW | LOW | SOME CONCERNS | LOW | LOW |

| **Reference** | | Chaiyasut *et al*., 2022 | Depommier *et al*., 2019 | Chaiyasut *et al*., 2021 | Palacios *et al*., 2021 | Lee *et al*., 2014 | Leber *et al*., 2012 | Kopp *et al*., 2023 |
| --- | --- | --- | --- | --- | --- | --- | --- | --- |
| Randomization | Sequence generation random | Y | Y | Y | Y | Y | Y | Y |
|  | Sequence concealment | Y | Y | Y | Y | PY | PY | PY |
|  | Baseline Differences | N | N | N | N | N | PN | N |
|  | Risk of Bias | LOW | LOW | LOW | LOW | LOW | LOW | LOW |
| Deviations | Participant Aware of Assignment | N | N | N | N | N | Y | N |
|  | Researcher Aware of Assignment | N | N | N | N | N | Y | N |
|  | Any Deviations | NA | NA | NA | NA | NA | N | NA |
|  | Deviations Impact | NA | NA | NA | NA | NA | NA | NA |
|  | Deviations Balanced | NA | NA | NA | NA | NA | NA | NA |
|  | Risk of Bias | LOW | LOW | LOW | LOW | LOW | LOW | LOW |
|  | Appropriate analysis | Y | N | Y | Y | NI | NI | Y |
|  | Appropriate Analysis Impact | NA | PN | NA | NA | N | N | NA |
|  | Risk of Bias | LOW | SOME CONCERNS | LOW | LOW | SOME CONCERNS | SOME CONCERNS | LOW |
| Missing Outcomes | Data Availability All Participants | Y | PN | Y | Y | Y | PY | PY |
|  | Non-bias by missing data | NA | PN | NA | NA | NA | NA | NA |
|  | Could Missingness Depend on Value | NA | PN | NA | NA | NA | NA | NA |
|  | Likely Missingness Depend | NA | NA | NA | NA | NA | NA | NA |
|  | Risk of Bias | LOW | LOW | LOW | LOW | LOW | LOW | LOW |
| Measurement | Method Inappropriate | N | N | N | N | N | N | N |
|  | Method Differ Between Groups | N | N | N | N | N | N | N |
|  | Assessors Awareness | NI | NI | NI | NI | NI | NI | N |
|  | Could Awareness Influence | PN | PN | PN | PN | PN | PN | NA |
|  | Likely Awareness Influence | NA | NA | NA | NA | NA | NA | NA |
|  | Risk of Bias | LOW | LOW | LOW | LOW | LOW | LOW | LOW |
| Reporting Bias | Analyzed with Pre-specified Plan | Y | Y | PY | Y | PY | Y | Y |
|  | Selected with Multiple Outcome | PN | PN | PN | N | PN | N | N |
|  | Selected with Multiple Analyses | PN | PN | PN | PN | PN | N | N |
|  | Risk of Bias | LOW | LOW | LOW | LOW | LOW | LOW | LOW |
| Overall Risk | Risk of Bias Judgement | LOW | LOW | LOW | LOW | LOW | LOW | LOW |

Abbreviations: Y, yes; PY, probably yes; N, no; PN, probably no; NI, no information; NA, not applicable

Quality of included human parallel arm trials was assessed using the Revised Cochrane risk-of-bias tool for randomized trials (RoB 2). The full list of questions in each domain can be found in the full guidance document for RoB 2.

**Supplemental Table 5.** Assessment of Study Quality and Risk of Bias of Crossovers According to Cochrane RoB2 Tool

| Pražnikar *et al*., 2020 | | | | | |
| --- | --- | --- | --- | --- | --- |
| Randomization | | | | | |
| 1.1 Sequence generation random | 1.2 Sequence concealment | 1.3 Baseline Differences | Risk of Bias |  |  |
| Y | Y | N | LOW |  |  |
| Deviations | | | | | |
| 2.1 Participant Aware of Assignment | 2.2 Researcher Aware of Assignment | 2.3 Any Deviations | 2.4 Deviations Impact | 2.5 Deviations Balanced | Risk of Bias |
| N | N | NA | NA | NA | LOW |
| 2.6 Appropriate analysis | 2.7 Appropriate Analysis Impact | Risk of Bias |  |  |  |
| Y | NA | LOW |  |  |  |
| Missing outcomes | | | | | |
| 3.1 Data Availability All Participants | 3.2 Non-bias by missing data | 3.3 Could Missingness Depend on Value | 3.4 Likely Missingness Depend | Risk of Bias |  |
| Y | NA | NA | NA | LOW |  |
| Measurement | | | | | |
| 4.1 Method Inappropriate | 4.2 Method Differ Between Groups | 4.3 Assessors Awareness | 4.4 Could Awareness Influence | 4.5 Likely Awareness Influence | Risk of Bias |
| N | N | N | NA | NA | LOW |
| Reporting bias | | | | | |
| 5.1 Analysed with Pre-specified Plan | 5.2 Selected with Multiple Outcome | 5.3 Selected with Multiple Analyses | 5.4 Selected with Carryover | Risk of Bias |  |
| Y | N | PN | N | LOW |  |
| Period and Crossover | | | | Overall Risk | |
| S.1 Equal Number of Participants | S.2 Period effects accounted for | S.3 Sufficient Washout | Risk of Bias | Risk of Bias Judgement |  |
| Y | NA | PY | LOW | LOW |  |

Abbreviations: Y, yes; PY, probably yes; N, no; PN, probably no; NI, no information; NA, not applicable

Quality of included human crossover trials was assessed using the Revised Cochrane risk-of-bias tool for randomized crossover trials (RoB 2). The full list of questions in each domain can be found in the full guidance document for RoB 2 additional considerations for crossover trials.
